# The Association Between Dietary Inflammatory Index and Cognitive Performance in Older Adults Aged 60 Years and Older

**DOI:** 10.3389/fnut.2022.748000

**Published:** 2022-04-12

**Authors:** Wenlei Song, Yijun Feng, Zonglin Gong, Changwei Tian

**Affiliations:** ^1^Department of Disease Control, Kunshan Centers for Disease Control and Prevention, Kunshan, China; ^2^Department of Nursing, Zhouzhuang People’s Hospital, Kunshan, China

**Keywords:** dietary inflammatory index, dose-response analysis, national health and nutrition examination survey, older adults, cognitive performance

## Abstract

**Background:**

Neuroinflammation has been linked to the development of cognitive performance. Epidemiological evidence on dietary inflammatory potential and cognitive performance is scarce. We evaluated the association between dietary inflammatory index (DII) and cognitive performance in older adults.

**Methods:**

This study included adults aged 60 years or older from the 2011–2014 National Health and Nutrition Examination Survey. The DII scores were calculated based on 27 nutritional parameters. Cognitive performance was assessed with four cognitive tests: the Digit Symbol Substitution Test (DSST, *n* = 2,780), the Consortium to Establish a Registry for Alzheimer’s Disease Word Learning (CERAD-WL, *n* = 2,859) and Delayed Recall (CERAD-DR, *n* = 2,857), and the Animal Fluency (AF, *n* = 2,844) tests. Restricted cubic splines and logistic regression were adopted to assess the associations.

**Results:**

Comparing the highest to lowest tertile of DII scores, the odds ratio (95% CI) of lower cognitive functioning was 1.97 (1.08–3.58) [*P*-trend = 0.02, per 1 unit increment: 1.17 (1.01–1.38)] on DSST, 1.24 (0.87–1.76) [*P*-trend = 0.24, per 1 unit increment: 1.09 (0.96–1.23)] on CERAD-WL, 0.93 (0.57–1.51) [*P*-trend = 0.74, per 1 unit increment: 1.02 (0.87–1.20)] on CERAD-DR, and 1.76 (1.30–2.37) [*P*-trend < 0.01, per 1 unit increment: 1.17 (1.05–1.29)] on AF. The above-mentioned associations were observed in both men and women. In non-linear dose–response analysis, the association between DII and lower cognitive functioning was not significant at lower DII scores up to 3.0, after which the association was significant and the curve rose steeply.

**Conclusion:**

Higher DII is associated with lower scores on DSST and AF tests in older adults.

## Introduction

Globally, the prevalence of cognitive impairment is 19.0% among adults aged 50 years and older (1). By 2050, the dementia prevalence is projected to triple worldwide and double in Europe, and the number of people living with dementia will increase to 152 million (2, 3). Twelve modifiable risk factors for cognitive impairment, including less education, obesity and depression, account for around 40% of worldwide dementias, and the prevention potential could be higher in middle-income and low-income countries where the prevalence of cognitive impairment is increasing more rapidly (3). Nutrition is an important modifiable risk factor of cognitive impairment, and use of nutrition assessment with food groups or dietary patterns is important than individual nutrients because the cumulative effects of nutrients should be considered (4).

The dietary inflammatory index (DII) has been proposed to assess the inflammatory potential of the diet, and is associated with systemic inflammatory markers including tumor necrosis factor (TNF), interleukins, and C-reactive protein (5, 6). Some inflammatory molecules can cross the blood-brain barrier elevating neuroinflammation and, consequently, compromising cognitive functions (7–9). Neuroinflammation has a vital role in the pathogenesis of cognitive impairment, and patients with Alzheimer’s disease showed increased levels of inflammatory markers including interleukins and TNF (10). However, epidemiological studies on the association between DII and cognitive impairment are scarce (11). The limited findings suggested that higher DII scores were associated with higher risk of cognitive impairment (12, 13) and declined memory function (14) in older adults. Based on the above-mentioned findings, we hypothesize that diets with higher pro-inflammatory potential are linked to cognitive impairment in older adults. Therefore, this study aimed to (1) explore the association between DII and cognitive performance in older adults; (2) explore the non-linear dose-response relationship between DII and cognitive performance [biological gradient is an essential component for determining an association (15)]; and (3) determine whether the association is independent of other key covariates. In addition, the limited findings available suggested that the association between diets with higher pro-inflammatory potential and risk of cognitive impairment was evident in women (12, 13), while no association was observed in men (13). Therefore, in this study, we also conducted stratified analyses by sex to explore the potential interaction effect of sex and DII on cognitive performance.

## Materials and Methods

### Participants

This cross-sectional study combined data from the 2011 to 2012 and 2013 to 2014 National Health and Nutrition Examination Survey (NHANES) cycles, because these two cycles specifically assessed cognitive performance for individuals aged 60 years and older. The combined data is also a nationally representative sample. Subjects who did not participant in cognitive performance tests (*n* = 167) and whose food parameters to calculate DII scores are missing (*n* = 564) were excluded from this study.

### Cognitive Performance Tests

Individuals aged 60 years and older were eligible. Cognitive performance was assessed with four cognitive tests: the Digit Symbol Substitution Test (DSST), the Consortium to Establish a Registry for Alzheimer’s Disease Word Learning (CERAD-WL) and Delayed Recall (CERAD-DR), and the Animal Fluency (AF) tests. The DSST was conducted using a paper form that has a key at the top containing 9 numbers paired with symbols. Participants had 2 min to copy the corresponding symbols in the 133 boxes that adjoin the numbers. The score is the total number of correct matches. A DSST cutoff of < 34 was used to classify lower cognitive functioning (16). In addition, we also used DSST < 40 as a cutoff in sensitivity analysis (17). There are three consecutive learning trials in the CERAD-WL. Participants were instructed to read aloud 10 unrelated words, and the order of the 10 words was changed in each of the three learning trials. A CERAD-WL cutoff of < 17 was used to classify lower cognitive functioning (16). For the AF test, participants were asked to name as many animals as possible in 1 min, and a point was given for each named animal. An AF cutoff of < 14 was used to classify lower cognitive functioning (16). In CERAD-DR, participants were asked to recall the 10 words approximately 8–10 min later from the start of the CERAD-WL and a CERAD-DR cutoff of < 5 was used to classify lower cognitive functioning (16).

### Dietary Inflammatory Index Scores

In NHANES, the DII scores were calculated with 27 food parameters, including alcohol, β-carotene, vitamin A, vitamin B_1_, vitamin B_2_, vitamin B_6_, vitamin B_12_, niacin, folic acid, vitamin C, vitamin D, vitamin E, monounsaturated fatty acids, n-3 fatty acids, n-6 fatty acids, protein, polyunsaturated fatty acids, saturated fatty acids, carbohydrate, total fat, fiber, caffeine, cholesterol, iron, magnesium, selenium, and zinc. The validity and the ability to predict inflammation of the DII calculated with the food parameters in NHANES has been shown (18–20). Among the 27 food parameters, saturated fat and total fat have the maximum pro-inflammatory effects, while fiber, β-Carotene, magnesium, n-3 fatty acids, and vitamins (A, C, D, and E) have the maximum anti-inflammatory effects (5).

The details of DII development have been described elsewhere (5), and the standard mean and standard deviation for each parameter included in the DII from the global composite data set are also available (5). First, Z scores were generated by subtracting the standard mean from the actual individual exposure and dividing this by the standard deviation for each parameter from the representative world database. These Z scores were then converted to percentile scores, which were doubled and then 1 is subtracted. The respective inflammatory effect score was then multiplied by the centered percentile value, and summed to create the DII scores for each individual. Data from a single 24-h dietary recall interviews was used to calculate the DII scores, which has been validated (i.e., the correlation with inflammatory markers) in previous studies (21, 22). We also performed a sensitivity analysis using the means of two 24-h dietary recall interviews.

### Covariates

In this study, the included covariates are as follows (11): sex, age (60–64, 65–69, 70–74, 75–79, and ≥ 80), race/ethnicity (Non-Hispanic White, Mexican American, Non-Hispanic Black, Other Hispanic, Other Race), body mass index (< 25 kg/m^2^, 25–29 kg/m^2^, ≥ 30 kg/m^2^), marital status (never married, married, others), education (≤ 11th grade, high school graduate, some college or AA degree, college graduate or above), poverty-income ratio (< 1, 1–2, > 2), smoking (current smoker, former smoker, never smoker), number of chronic diseases (0, 1, 2, ≥ 3), depression (major depression, others), health status (fair/poor, excellent/very good/good) and energy intake.

Chronic diseases included: hypertension, stroke, myocardial infarction, coronary heart disease, angina, arthritis, emphysema, asthma, chronic bronchitis, and diabetes mellitus. The Current Health Status section in the NHANES provides personal interview data on general health condition, which was assessed with the question: “Would you say your health in general is poor, fair, good, very good, or excellent”? Depression was measured using the Patient Health Questionnaire, a nine-item screening instrument that asked questions about the frequency of symptoms of depression over the past 2 weeks. A total score is based on the sum of the points in each item ranging from 0 to 27, and major depression was defined if the scores were 10 or higher (23). Diabetes was defined by a hemoglobin A1c level of ≥ 6.5%, a fasting plasma glucose level of ≥ 126 mg/dL, or 2-h plasma glucose of ≥ 200 mg/dL (24), or a previous diagnosis of diabetes. Hypertension was defined by a systolic blood pressure level of ≥ 130 mmHg, or a diastolic blood pressure level of ≥ 80 mmHg, or taking antihypertensive medicine currently (25). The mean values of three measurements of systolic blood pressure and diastolic blood pressure were used in this analysis. Other chronic diseases were defined by the question: “Has a doctor or other health professional told you that you had [diseases]”?

### Statistical Analysis

Logistic regression model was used to assess the association between DII and cognitive performance. Given the relatively small number of participants, subjects were classified into tertiles according to their DII scores. Compared with subjects in tertile 1, the odds ratios (ORs) (95% CIs) of scoring low on cognitive performance tests for subjects in tertile 2 and tertile 3 were calculated. We calculated three different logistic regression models. Model 1 was adjusted for sex, age and race/ethnicity. Model 2 was adjusted for covariates in model 1, and also education, body mass index, marital status, poverty-income ratio and smoking. Model 3 was adjusted for covariates in model 2, and also chronic diseases, health status, depression and energy intake. Tests for trends across tertiles were conducted by modeling the DII scores as a continuous variable using the median values of DII scores in each tertile, respectively. In addition, we also calculated the OR (95%CI) of scoring low cognitive performance tests for each 1 unit increment in DII scores. Restricted cubic spline functions are powerful tools to characterize dose-response associations between continuous exposures and health outcomes (26). The potential non-linear dose-response relationship between DII and cognitive performance was examined by modeling DII scores using restricted cubic splines, and we used three knots at 25, 50, and 75% percentiles of the DII scores distribution (26). The departure from a linear relationship was considered significant if the coefficient of the second spline is not equal to 0 (26). The median level in tertile 1 of DII scores was used as the reference in the dose-response analysis (27–29). Stratified analysis by sex was conducted, and the cross-product term of DII with sex was included in the model to test the interaction. Appropriate strata, cluster, and weights were considered in all analyses as suggested by NHANES, and we computed the multi-year sample weight by dividing the 2-year sample weights by 2. We also performed a sensitivity analysis in which participants with 3 or more number of chronic diseases were excluded. STATA version 12.0 was used in this analysis, and the result was considered statistically significant if the *P*-value was ≤ 0.05 in the analysis.

## Results

### Study Sample

A total of 2,901 adults aged 60 years and older were included in this study. The number of participants included in each analysis was 2,780 for DSST, 2,859 for CERAD-WL, 2,857 for CERAD-DR, and 2,844 for AF, respectively. The weighted prevalence of lower cognitive functioning was 14.09% for DSST, 22.58% for CERAD-WL, 25.17% for CERAD-DR, and 21.86% for AF, respectively. The mean (range) of DII scores was 2.02 (−3.93, 5.20) in the sample. Compared with participants in tertile 1 of DII scores, participants with higher DII scores showed higher prevalence of lower cognitive functioning on DSST, CERAD-WL and AF. The differences across DII groups were significant for all covariates (*P* < 0.01), except for age (*P* = 0.15). Detailed characteristics of the study participants are shown in Table 1.

**TABLE 1:** Population characteristics by dietary inflammatory index scores.

Characteristics	Overall	Tertile 1	Tertile 2	Tertile 3	*P*-value^a^
DII score	2.02 (1.46)	0.36 (0.95)	2.13 (0.36)	3.54 (0.57)	
Age, year	69.61 (6.83)	69.29 (6.76)	69.89 (6.96)	69.66 (6.76)	0.15
Male, %	49.09	59.56	46.87	41.08	<0.01
DSST < 34, %	14.09	8.70	12.25	23.15	<0.01
CERAD-WL < 17, %	22.58	19.36	20.45	29.12	<0.01
CERAD-DR < 5, %	25.17	24.07	23.56	28.62	0.06
AF < 14, %	21.86	14.75	21.95	30.63	<0.01
Number of chronic diseases					<0.01
0	6.86	9.20	7.20	4.26	
1	27.89	28.94	29.54	25.25	
2	31.44	33.44	30.38	30.53	
≥3	33.82	28.42	32.88	39.96	
Education, %					<0.01
<11th grade	26.49	4.95	6.42	6.19	
High school graduate	23.11	24.74	25.67	29.70	
Some college or AA degree	27.87	30.34	31.94	30.57	
College graduate or above	22.53	39.97	35.97	33.54	
Race/Hispanic origin (%)					<0.01
Mexican American	8.82	9.40	8.77	8.32	
Other Hispanic	10.10	9.51	10.44	10.34	
Non-Hispanic White	47.78	50.57	50.73	42.19	
Non-Hispanic Black	24.37	19.64	21.50	31.74	
Other Race	8.93	10.87	8.56	7.40	
Marital status, %					<0.01
Never married	5.94	6.48	4.49	6.81	
Married	54.52	60.82	54.23	48.68	
Others	39.54	32.71	41.27	44.51	
Ratio of family income to poverty (%)					<0.01
<1	17.64	13.58	16.23	22.97	
1–2	29.71	25.36	31.20	32.53	
2–5	52.65	61.05	52.57	44.51	
General health condition					<0.01
Excellent/very good/good	70.62	77.47	71.25	63.38	
Fair/poor	29.38	22.53	28.75	36.62	
Major depression					<0.01
Depression screen < 10	90.99	92.40	91.97	88.64	
Depression screen ≥ 10	9.01	7.60	8.03	11.36	
Smoking status (%)					<0.01
Current smoker	12.48	9.40	11.48	16.43	
Former smoker	37.95	44.20	37.58	32.25	
Never smoker	49.57	46.39	50.94	51.32	
Energy intake (kcal)	1827.92 (803.06)	2404.94 (860.89)	1808.98 (571.96)	1286.27 (494.00)	<0.01

*Means (SDs) are shown for continuous variables.*

*DSST, Digit Symbol Substitution Test; CERAD-WL, the Consortium to Establish a Registry for Alzheimer’s Disease Word Learning; CERAD-DR, the Consortium to Establish a Registry for Alzheimer’s Disease Delayed Recall; AF, the Animal Fluency.*

*^a^P-values were calculated with Chi-square test for categorical variables and ANOVA test for continuous variables.*

### Logistic Regression

In model 1 and model 2, higher DII scores were associated with scoring low on DSST, CERAD-WL and AF. The magnitude of the associations with DSST and AF was attenuated in model 3, and the association with CERAD-WL was not significant in model 3. No association was found between DII scores and CERAD-DR in there statistical models. In model 3, comparing the highest to lowest tertile of DII scores, the OR (95% CI) of lower cognitive functioning was 1.97 (1.08–3.58) [*P*-trend = 0.02, per 1 unit increment: 1.17 (1.01–1.38)] on DSST, 1.24 (0.87–1.76) [*P*-trend = 0.24, per 1 unit increment: 1.09 (0.96–1.23)] on CERAD-WL, 0.93 (0.57–1.51) [*P*-trend = 0.74, per 1 unit increment: 1.02 (0.87–1.20)] on CERAD-DR, and 1.76 (1.30–2.37) [*P*-trend < 0.01, per 1 unit increment: 1.17 (1.05–1.29)] on AF. Overall, the above-mentioned findings were similar in men and women, and the interactions with sex were not significant in all analyses (all *P* > 0.05). The detailed results are shown in Tables 2, 3.

**TABLE 2:** Odds ratios (95% confidence intervals) of lower cognitive functioning by dietary inflammatory index scores.

Cognitive test	Tertile 1	Tertile 2	Tertile 3	*P*-trend	Per 1 unit increment
**DSST < 34**					
Model 1	1.00	1.64 (1.31–2.04)**	3.47 (2.27–5.31)**	<0.01	1.40 (1.22–1.60)**
Model 2	1.00	1.33 (0.98–1.81)	2.93 (1.82–4.70)**	<0.01	1.30 (1.13–1.50)**
Model 3	1.00	1.08 (0.74–1.57)	1.97 (1.08–3.58)*	0.02	1.17 (1.01–1.38)*
**CERAD-WL < 17**					
Model 1	1.00	1.13 (0.85–1.52)	1.93 (1.46–2.54)**	<0.01	1.22 (1.13–1.32)**
Model 2	1.00	1.01 (0.72–1.43)	1.50 (1.13–2.01)**	<0.01	1.14 (1.05–1.24)**
Model 3	1.00	0.92 (0.65–1.29)	1.24 (0.87–1.76)	0.24	1.09 (0.96–1.23)
**CERAD-DR < 5**					
Model 1	1.00	1.06 (0.73–1.53)	1.29 (0.90–1.85)	0.18	1.10 (0.99–1.22)
Model 2	1.00	0.96 (0.66–1.39)	1.09 (0.79–1.50)	0.64	1.06 (0.96–1.16)
Model 3	1.00	0.87 (0.54–1.40)	0.93 (0.57–1.51)	0.74	1.02 (0.87–1.20)
**AF < 14**					
Model 1	1.00	1.65 (1.19–2.29)**	2.23 (1.67–2.97)**	<0.01	1.27 (1.17–1.38)**
Model 2	1.00	1.46 (1.03–1.07)*	1.82 (1.35–2.44)**	<0.01	1.19 (1.09–1.29)**
Model 3	1.00	1.41 (1.01–1.95)*	1.76 (1.30–2.37)**	<0.01	1.17 (1.05–1.29)**

*Model 1 was adjusted for age, sex and race/ethnicity.*

*Model 2 was adjusted for covariates in model 1, and also body mass index, poverty-income ratio, education, marital status and smoking.*

*Model 3 was adjusted for covariates in model 2, and also chronic disease, health status, depression and energy intake.*

*P < 0.05, **P < 0.01.

*DSST, Digit Symbol Substitution Test; CERAD-WL, the Consortium to Establish a Registry for Alzheimer’s Disease Word Learning; CERAD-DR, the Consortium to Establish a Registry for Alzheimer’s Disease Delayed Recall; AF, the Animal Fluency.*

**TABLE 3:** Odds ratios (95% confidence intervals) of lower cognitive functioning by tertiles of dietary inflammatory index score and by sex.

Cognitive test	Men					Women				
		
	Tertile 1	Tertile 2	Tertile 3	*P*-trend	Per 1 unit increment	Tertile 1	Tertile 2	Tertile 3	*P*-trend	Per 1 unit increment
**DSST < 34**										
Model 1	1.00	1.18 (0.74–1.90)	3.37 (1.99–5.71)**	<0.01	1.40 (1.20–1.63)**	1.00	1.51 (0.88–2.58)	2.85 (1.76–4.62)**	<0.01	1.39 (1.19–1.63)**
Model 2	1.00	0.96 (0.53–1.74)	2.59 (1.29–5.21)**	0.01	1.27 (1.07–1.52)**	1.00	1.24 (0.68–2.27)	2.59 (1.52–4.44)**	<0.01	1.33 (1.12–1.58)**
Model 3	1.00	0.74 (0.39–1.37)	1.97 (1.06–3.95)*	0.04	1.15 (1.02–1.30)*	1.00	1.01 (0.51–1.98)	1.92 (1.00–3.85)*	0.05	1.19 (1.01–1.41)*
**CERAD-WL < 17**										
Model 1	1.00	1.08 (0.76–1.54)	2.06 (1.31–3.23)**	<0.01	1.22 (1.08–1.37)**	1.00	1.30 (0.87–1.94)	1.96 (1.39–2.76)**	<0.01	1.22 (1.09-1.37)**
Model 2	1.00	1.01 (0.67–1.52)	1.62 (1.10–2.39)*	0.02	1.13 (1.01-1.26)*	1.00	1.15 (0.72–1.84)	1.69 (1.11–2.58)*	0.02	1.17 (1.03–1.34)*
Model 3	1.00	0.96 (0.63–1.45)	1.49 (0.80–2.74)	0.23	1.10 (0.91–1.31)	1.00	1.03 (0.63–1.70)	1.32 (0.80–2.18)	0.26	1.07 (0.90–1.27)
**CERAD-DR < 5**										
Model 1	1.00	0.91 (0.56–1.50)	0.93 (0.54–1.59)	0.76	1.02 (0.91–1.15)	1.00	1.27 (0.92–1.74)	1.69 (1.17–2.42)**	<0.01	1.22 (1.07–1.39)**
Model 2	1.00	0.80 (0.47–1.36)	0.77 (0.47–1.26)	0.27	0.97 (0.87–1.08)	1.00	1.25 (0.82–1.92)	1.55 (0.99–2.43)	0.05	1.20 (1.03–1.39)*
Model 3	1.00	0.75 (0.43–1.32)	0.73 (0.42–1.27)	0.24	0.97 (0.83–1.13)	1.00	1.06 (0.62–1.80)	1.11 (0.55–2.23)	0.76	1.09 (0.85–1.40)
**AF < 14**										
Model 1	1.00	1.69 (1.09–2.61)*	2.17 (1.44–3.27)**	<0.01	1.28 (1.14–1.44)**	1.00	1.30 (0.91–1.85)	2.12 (1.49–3.00)**	<0.01	1.28 (1.13–1.43)**
Model 2	1.00	1.53 (0.96–2.45)	1.66 (1.04–2.65)*	0.03	1.18 (1.04–1.33)*	1.00	1.17 (0.76–1.81)	1.95 (1.35–2.83)**	<0.01	1.22 (1.07–1.39)**
Model 3	1.00	1.63 (1.03–2.59)*	2.09 (1.18–3.71)*	0.04	1.28 (1.08–1.50)**	1.00	1.02 (0.62–1.69)	1.56 (1.03–2.31)*	0.04	1.12 (1.01–1.24)*

*Model 1 was adjusted for age, sex and race/ethnicity.*

*Model 2 was adjusted for covariates in model 1, and also body mass index, poverty-income ratio, education, marital status and smoking.*

*Model 3 was adjusted for covariates in model 2, and also chronic disease, health status, depression and energy intake.*

*P < 0.05, **P < 0.01.

*DSS, Digit Symbol Substitution Test; CERAD-WL, the Consortium to Establish a Registry for Alzheimer’s Disease Word Learning; CERAD-DR, the Consortium to Establish a Registry for Alzheimer’s Disease Delayed Recall; AF, the Animal Fluency.*

The above-mentioned findings were similar in a sensitivity analysis after excluding participants with 3 or more number of chronic diseases (29.63%), who may change their dietary habits (Supplementary Table 1). Similar results were also found in a sensitivity analysis using the means of two 24-h dietary recall interviews to calculate the DII scores, and in a sensitivity analysis using DSST < 40 as a cutoff. In a sensitivity analysis with DSST score < 40 as a cutoff, and comparing the highest to lowest tertile of DII scores, the OR (95% CI) of scoring low on DSST was 1.97 (1.33–2.94) [*P*-trend < 0.01, per 1 unit increment: 1.20 (1.06–1.35)] in model 3. In addition, the results did not change materially when the covariates of BMI and age were included as continuous variables in the model.

### Dose-Response Analysis

In dose-response analysis, the median level in tertile 1 of DII (0.36) was used as the reference.

**FIGURE 1:**
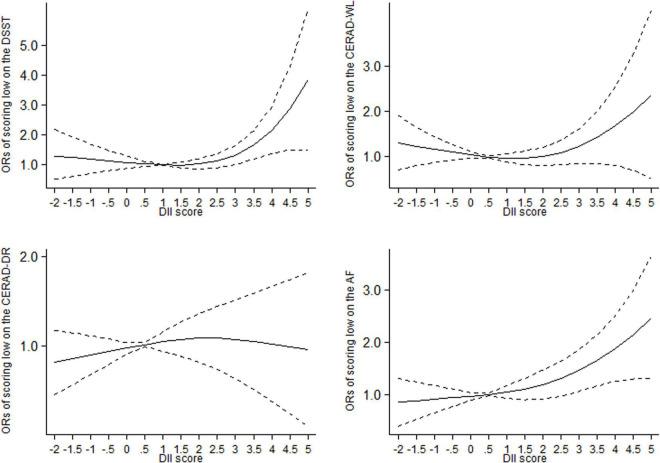
Dose-response relationships between dietary inflammatory index (DII) and the odds of scoring low on the DSST, CERAD-DR, CERAD-WL and AF in older adults, respectively. The middle solid line represents the estimated odds ratio, and the upper and lower short-dash line represents its 95% confidence interval. DSST: Digit Symbol Substitution Test. CERAD-WL: the Consortium to Establish a Registry for Alzheimer’s Disease Word Learning, CERAD-DR: the Consortium to Establish a Registry for Alzheimer’s Disease Delayed Recall, AF: the Animal Fluency.

The departure from a linear relationship was significant for the association between DII and scoring low on DSST (*P*_*for non–linearity*_ = 0.02) and CERAD-WL (*P*_*for non–linearity*_ = 0.04), respectively, while the departure from a linear relationship was not significant for the association between DII and scoring low on CERAD-DR (*P*_*for non–linearity*_ = 0.40) and AF (*P*_*for non–linearity*_ = 0.27), respectively. In non-linear dose–response analysis, the association between DII and cognitive performance was not significant at lower DII scores up to 3.0, after which the association was significant and the curve rose steeply (Figure 1). At DII scores of 3.0, the OR (95% CI) of scoring low on DSST was 1.35 (1.00–1.71), 1.22 (0.85–1.60) for scoring low on CERAD-WL, 1.07 (0.63–1.51) for scoring low on CERAD-DR, and 1.47 (1.07–1.86) for scoring low on AF, respectively.

## Discussion

In this study, higher DII scores were associated with scoring low on DSST and AF in older adults, and the association was consistent in men and women. The associations between DII and scoring low on DSST and AF were attenuated after adjustment for other key covariates. The above association was dose-dependent, and DII scores of above 3.0 were associated with scoring low on DSST and AF.

Epidemiological evidence on DII and cognitive impairment is limited. A recent review summarized the evidence from seven epidemiological studies on DII and cognition, frailty and disabilities in older adults (11), and the findings showed that DII was significantly associated with cognition (11). The association between DII and risk of disability was also observed in the NHANES (28). Three studies have explored the associations between DII and memory, cognitive decline and risk of dementia (12–14). In The Women’s Health Initiative Memory Study (*n* = 7,085), over an average of 9.7 years, the adjusted hazard ratios of mild cognitive impairment/dementia compared to the group with the lowest inflammatory potential (group 1) were 1.01 (0.86–1.20) for group 2 (DII scores: −2 to 0), 0.99 (0.82–1.18) for group 3 (DII scores: 0–2) and 1.27 (1.06–1.52) for group 4 (DII scores: > 2) (12), suggesting that DII scores of above 2 were significantly associated with cognitive impairment. A cross-sectional study from an older adult Korean population (*n* = 239) showed that the adjusted ORs of cognitive impairment comparing lower energy-adjusted DII scores to the higher scores were 2.57 (0.40–16.63) for tertile 2 and 6.32 (1.18–33.78) for tertile 3 (13), suggesting that the association was also dose-dependent. In addition, using multivariable linear regression, Frith et al. showed that higher DII scores were inversely associated with all memory parameters (14). Therefore, our findings are comparable with the above-mentioned results with different study designs and populations.

As a literature-derived dietary index, the DII is developed to predict inflammation, and has been adopted to explore the associations between dietary inflammatory potential and risk of a wide range of non-communicable diseases (30). Higher DII scores reflect a more pro-inflammatory diet, while the lower scores indicate a more anti-inflammatory diet. The associations between higher DII scores and increased levels of various inflammatory markers have been observed in different populations (5, 6, 19, 31). The inflammatory markers can then cross the blood-brain barrier and subsequently elevate the neuroinflammation, which exert a prominent effect on the pathogenesis of neurodegenerative disease (7–10, 32, 33). Neuroinflammation is marked by the production of several pro-inflammatory cytokines and the detailed mechanisms have been summarized in the recent well-conducted review (10). Briefly, over-expression of pro-inflammatory molecules can cause synaptic dysfunction, neuronal death and inhibition of neurogenesis (10). In addition, the inflammatory pathway is also proposed as an important bridge between gut microbiota and neurodegenerative disease (33). In this study, higher DII scores were associated with DSST and AF, but were not significantly associated with the CERAD-WL and CERAD-DR after adjusting for other covariates. The DSST relies on working memory, processing speed and sustained attention, and is adopted as a tool for assessing frontal lobe executive function. The AF test examines verbal semantic fluency. The CERAD test assesses new learning, immediate memory and delayed memory. With regard to specific cognitive domains, results from our study are generally consistent with previous studies in which inflammation was significantly associated with working memory, processing speed, verbal fluency, attention and executive function (34, 35), while the associations with learning and recall were not significant (35). These findings suggested that higher DII scores might have different effects on domain-specific cognitive function.

Strengths of this study included that this is a nationally representative sample of US older adults, and both logistic regression and dose-response analysis were conducted. In addition, a wide range of covariates was considered in this analysis. There are several potential limitations. First, this analysis cannot determine the causality as this is a cross-sectional study. However, similar findings were found in sensitivity analysis excluding participants with more number of chronic diseases who may change their dietary habits. In addition, the association between DII and cognitive performance does meet several aspects for deducing causation (15): (1) strength—the strength of the observed association between DII and potential cognitive impairment is not negligible; (2) consistency—the positive association between DII and potential cognitive impairment is consistent in men and women; (3) temporality—although the temporal relationship between DII and potential cognitive impairment cannot not be assessed in this study, the only prospective cohort study found that higher DII scores could increase the risk of cognitive impairment (12); (4) biological gradient—the dose–response relationship suggested that the association was dose-dependent. In this study, the association between DII and cognitive performance was not significant at lower DII scores, while the association was significant and the curve rose steeply at higher DII scores. The relationships are comparable to previous studies on DII and other health outcomes like cancers (36), cardiometabolic diseases mortality (37), chronic kidney disease (38) and disability in older adults (28), although the “threshold DII values” may differ across different health effects; (5) plausibility and coherence—the findings available suggest that it is biologically plausible for causality in that diets with higher pro-inflammatory potential could increase the risk of cognitive impairment. However, the Bradford Hill ultimate criteria for causation, i.e., the findings from randomized trials are lacking. Second, bias such as misclassification of diets is of concern in observational studies. However, non-differential misclassification should have weakened the association. Third, based on the available NHANES data, only 27 of the original 45 parameters were included to calculate DII. Therefore, the calculated DII might underestimate the participant’s true DII, and as a result, the observed association between DII and cognitive performance might be underestimated. However, the validity and the ability to predict inflammation of the DII calculated with the food parameters in NHANES has been shown (18–20). Finally, potential cognitive impairment was assessed by cognitive performance tests rather than clinical examination. However, cognitive performance tests are useful to explore the associations between cognitive function and health outcomes and risk factors in NHANES (16, 39, 40).

In conclusion, higher DII is associated with lower scores on DSST and AF tests in older adults in both men and women, and the associations were dose-dependent. The results deserve to be confirmed in other populations and in prospective cohort studies.

## Data Availability Statement

Publicly available datasets were analyzed in this study. This data can be found here: The datasets for this study can be found in the NHANES: https://www.cdc.gov/nchs/nhanes/.

## Ethics Statement

The survey protocol was approved by the Research Ethics Review Board at the National Center for Health Statistics. Ethical approval for this study is deemed exempt because this study uses publically available secondary data. The patients/participants provided their written informed consent to participate in this study.

## Author Contributions

WS and CT: conceptualization, data curation, formal analysis, investigation, methodology, resources, and software. WS and YF: roles and writing—original draft. CT: writing—review and editing, supervision. All authors read and approved the final manuscript.

## Conflict of Interest

The authors declare that the research was conducted in the absence of any commercial or financial relationships that could be construed as a potential conflict of interest.

## Publisher’s Note

All claims expressed in this article are solely those of the authors and do not necessarily represent those of their affiliated organizations, or those of the publisher, the editors and the reviewers. Any product that may be evaluated in this article, or claim that may be made by its manufacturer, is not guaranteed or endorsed by the publisher.
