# Supplementary material for: The Association Between Dietary Inflammatory Index and Cognitive Performance in Older Adults Aged 60 Years and Older
Source: Front Nutr. 2022 Apr 12;9:748000. doi: 10.3389/fnut.2022.748000 (PMC9039302; doi:10.3389/fnut.2022.748000)
Supplement: Supplementary file 1 [file Table_1.DOCX]

Supplementary Table 1 Odds ratios (95% confidence intervals) of cognitive impairment by tertiles of dietary inflammatory index scores in sensitivity analysis excluding participants with 3 or more number of chronic diseases

| Cognitive test | Tertile 1 | Tertile 2 | Tertile 3 | *P*-trend | Per 1 unit increment |
| --- | --- | --- | --- | --- | --- |
| DSST<34 |  |  |  |  |  |
| Model 1 | 1.00 | 1.14 (0.81-1.61) | 3.34 (1.88-5.95)** | <0.01 | 1.45 (1.18-1.77)** |
| Model 2 | 1.00 | 1.02 (0.68-1.54) | 2.89 (1.50-5.57)** | <0.01 | 1.37 (1.10-1.69)** |
| Model 3 | 1.00 | 0.76 (0.48-1.19) | 1.56 (1.01-2.41)* | 0.05 | 1.16 (1.01-1.34)* |
| CERAD-WL<17 |  |  |  |  |  |
| Model 1 | 1.00 | 1.16 (0.79-1.71) | 2.27 (1.49-3.46)** | <0.01 | 1.29 (1.16-1.44)** |
| Model 2 | 1.00 | 1.17 (0.72-1.92) | 1.92 (1.34-2.74)** | <0.01 | 1.23 (1.11-1.36)** |
| Model 3 | 1.00 | 1.06 (0.64-1.74) | 1.69 (0.86-2.61) | 0.15 | 1.12 (0.93-1.33) |
| CERAD-DR<5 |  |  |  |  |  |
| Model 1 | 1.00 | 0.92 (0.63-1.34) | 1.43 (0.90-2.26) | 0.16 | 1.14 (1.00-1.31) |
| Model 2 | 1.00 | 0.87 (0.57-1.32) | 1.21 (0.83-1.78) | 0.39 | 1.10 (0.98-1.23) |
| Model 3 | 1.00 | 0.80 (0.49-1.30) | 1.02 (0.60-1.74) | 1.00 | 1.06 (0.90-1.25) |
| AF<14 |  |  |  |  |  |
| Model 1 | 1.00 | 1.28 (0.90-1.82) | 2.34 (1.69-3.24)** | <0.01 | 1.25 (1.13-1.39)** |
| Model 2 | 1.00 | 1.18 (0.78-1.77) | 1.93 (1.36-2.73)** | <0.01 | 1.16 (1.04-1.30)* |
| Model 3 | 1.00 | 1.29 (0.83-1.97) | 2.30 (1.52-3.47)** | <0.01 | 1.20 (1.05-1.37)* |

Model 1 was adjusted for age, sex and race/ethnicity.

Model 2 was adjusted for covariates in model 1, and also body mass index, poverty-income ratio, education, marital status and smoking.

Model 3 was adjusted for covariates in model 2, and also chronic disease, health status, depression and energy intake.

*: *P*<0.05, **: *P*<0.01

DSST: Digit Symbol Substitution Test. CERAD-WL: the Consortium to Establish a Registry for Alzheimer’s Disease Word Learning, CERAD-DR: the Consortium to Establish a Registry for Alzheimer’s Disease Delayed Recall, AF: the Animal Fluency.
